# New Books

**Published:** 2005-07

**Apoptotic Pathways as Targets for Novel Therapies in Cancer and Other Diseases**

Marek Los, Spencer B. Gibson, eds.

New York:Springer-Verlag, 2005. 396 pp. ISBN: 0-387-23384-9, $149

**BioIndustry Ethics**

David Finegold, Cecile Bensimon, Abdallah Daar, Margaret Eaton, Beatrice Godard, Bartha Knoppers, Jocelyn Mackie, Peter Singer

Burlington, MA:Elsevier, 2005. 384 pp. ISBN: 0-12-369370-5, $34.95

**Chemistry and Safety of Acrylamide in Food**

Mendel Friedman, Don Mottram, eds.

New York:Springer-Verlag, 2005. 475 pp. ISBN: 0-387-23920-0, $169

**Chernobyl: Catastrophe, Consequences and Solutions**

Jim Smith, Nicholas A. Beresford

New York:Springer-Verlag, 2005. 200 pp. ISBN: 3-540-23866-2, $119

**Encyclopedic Reference of Immunotoxicology**

Hans-Werner Vohr, ed.

New York:Springer-Verlag, 2005. 730 pp. ISBN: 3-540-44172-7, $299

**Environmental Health: From Global to Local**

Howard Frumkin, ed.

San Francisco, CA:Jossey-Bass, 2005. 1,250 pp. ISBN: 0-7879-7383-1, $65

**Facilitating Interdisciplinary Research**

Committee on Facilitating Interdisciplinary Research, National Academy of Sciences, National Academy of Engineering, Institute of Medicine

Washington, DC:National Academies Press, 2005. 332 pp. ISBN: 0-309-09435-6, $42

**Handbook of Isoelectric Focusing and Proteomics**

David Garfin, Satinder Ahuja

Burlington, MA:Elsevier, 2005. 380 pp. ISBN: 0-12-088752-5, $215

**Integrative Physiology in the Age of Genomics and Proteomics**

Wolfgang Walz

Totowa, NJ:Humana Press, 2005. 280 pp. ISBN: 1-58829-315-7, $99.50

**Intelligent Bioinformatics: The Application of Artificial Intelligence Techniques to Bioinformatics Problems**

Edward Keedwell, Ajit Narayanan

Hoboken, NJ:John Wiley & Sons, 2005. 294 pp. ISBN: 0-470-02175-6, $110

**Metabolome Analyses: Strategies for Systems Biology**

Seetharaman Vaidyanathan, George G. Harrigan, Royston Goodacre, eds.

New York:Springer-Verlag, 2005. 405 pp. ISBN: 0-387-25239-8, $129

**Methods in Community-Based Participatory Research for Health**

Barbara A. Israel, Eugenia Eng, Amy J. Schultz, Edith A. Parker, eds.

Hoboken, NJ:John Wiley & Sons, 2005. 528 pp. ISBN: 0-7879-7562-1, $60

**Molecular Biology: Understanding the Genetic Revolution**

David Clark

Burlington, MA:Elsevier, 2005. 816 pp. ISBN: 0-12-175551-7, $94.95

**Molecular Diagnostics**

George Patrinos, Wilhelm Ansorge, eds.

Burlington, MA:Elsevier, 2005. 488 pp. ISBN: 0-12-546661-7, $99.95

**Proteomics in Cancer Research**

Daniel C. Liebler, Emanuel F. Petricoin, Lance A. Liotta, eds.

Hoboken, NJ:John Wiley & Sons, 2005. 202 pp. ISBN: 0-471-44476-6, $150

**Statistical Methods in Molecular Evolution**

Rasmus Nielsen, ed.

New York:Springer-Verlag, 2005. 520 pp. ISBN: 0-387-22333-9, $89.95

**The p53 Tumor Suppressor Pathway and Cancer**

Gerard P. Zambetti, ed.

New York:Springer-Verlag, 2005. 245 pp. ISBN: 0-387-24135-3, $139

